# *SNPsplit*: Allele-specific splitting of alignments between genomes with known SNP genotypes

**DOI:** 10.12688/f1000research.9037.2

**Published:** 2016-07-27

**Authors:** Felix Krueger, Simon R. Andrews

**Affiliations:** 1 Bioinformatics Group, The Babraham Institute, Cambridge, UK

**Keywords:** Allele-specific, SNP, N-masking, ASM, ASE, ASB, Allele

## Abstract

Sequencing reads overlapping polymorphic sites in diploid mammalian genomes may be assigned to one allele or the other. This holds the potential to detect gene expression, chromatin modifications, DNA methylation or nuclear interactions in an allele-specific fashion. SNPsplit is an allele-specific alignment sorter designed to read files in SAM/BAM format and determine the allelic origin of reads or read-pairs that cover known single nucleotide polymorphic (SNP) positions. For this to work libraries must have been aligned to a genome in which all known SNP positions were masked with the ambiguity base 'N' and aligned using a suitable mapping program such as Bowtie2, TopHat, STAR, HISAT2, HiCUP or Bismark. SNPsplit also provides an automated solution to generate N-masked reference genomes for hybrid mouse strains based on the variant call information provided by the Mouse Genomes Project. The unique ability of SNPsplit to work with various different kinds of sequencing data including RNA-Seq, ChIP-Seq, Bisulfite-Seq or Hi-C opens new avenues for the integrative exploration of allele-specific data.

## Introduction

Most functional NGS studies performed today still ignore the fact that many model organisms are diploid, and work on the averaged signal from the two alleles. However, a complete understanding of the biology of diploid organisms requires that the two alleles be measured separately. Allele-specific analysis of next-generation sequencing reads is becoming an important tool to identify events such as allele-specific expression of genes (ASE), allele-specific binding of transcription factors or histones (ASB) or allele-specific methylation (ASM). These techniques allow a more detailed investigation of the effects of genetic or epigenetic variation on genome regulation or studying parent of origin effects such as genomic imprinting or allelic imbalance.

There are two main use cases for the investigation of allele-specific events: If both parental genotypes are clean and known in advance, e.g. for defined crosses of inbred mouse strains, parent of origin specific effects can be studied by comparing the two parental genotypes. Alternatively, allele-specific analyses require the more complex procedure of whole genome haplotype reconstruction (e.g. as described in 1). For the purposes of this manuscript we will use the terms 'Allele 1’ or 'Allele 2’ to refer to the maternal or paternal genotype, respectively, or to a reference and alternative strain or genome if the distinction between maternal/paternal is not meaningful.

The detection of allele-specific events relies on the ability to distinguish the two alleles of a diploid organism, which can be accomplished by looking at reads covering heterozygous single nucleotide polymorphisms (SNPs), small insertions or deletions (InDels) or greater structural variations. While the allele-specific analysis of InDels has been found to be challenging ^2^, the use of SNPs to discriminate alleles is the most widely used approach because it allows for the maintenance of a common set of reference genome coordinates.

Several approaches have been taken to perform allele-specific alignments. The simplest is to align all reads to a single reference genome, but this introduces a bias as reads from the allele which is more similar to the reference are able to map more efficiently ^3^. Another approach involves the generation of two personalised genomes by incorporating known SNP positions (and possibly InDels) followed by an alignment to both genomes and finally a post-processing step to compute the union of the separate alignments (used in different flavours in 4– 6). This approach is slower as it requires two separate mapping steps, and can still result in allelic bias because reads from one allele might not map uniquely or to an incorrect location in one of the genomes ^3^. A more recent improvement ^7^ aims to reduce mapping biases by first aligning reads to the reference genome, then realigning reads that overlap SNP positions in all possible allele combinations and keeping only reads that align to the same position regardless of their genotypes - this reduces bias, but is computationally complex. Finally, the issue of bias can be tackled by masking polymorphic sites with the ambiguity nucleobase ’N’ (henceforth called ’N-masking’), performing a single alignment to the N-masked genome and then assigning reads based on the sequence found underneath the masked positions. The rationale for N-masking in allele-specific alignments is that the mapping bias towards the reference allele is eliminated and both alleles of the same read get placed in the same position in the genome equally well. N-masking the genome is a one-off exercise and this approach has the advantage of requiring only a single alignment to a reference which noticeably reduces the computational load. Despite the fact that N-masking effectively avoids allelic biases it may occasionally result in a minor loss of sensitivity when the density of N covered by a read is getting too high.

A requirement for N-masking is that SNP positions are known, e.g. via a public resource like the Mouse Genomes Project which provides high quality variant calls for a large number of mouse strains ^8^ (hosted at http://www.sanger.ac.uk/science/data/mouse-genomes-project). If the genotype is not known, SNP positions may be called from the data itself, or from genome re-sequencing performed in parallel. The quality of the genotype calls is crucial for allele assignment, so the genotype data needs to be collected carefully and quality control and filtering is required to avoid biases and false positive hits ^9^. Further downstream analysis of allele-specific data is highly dependent on the experiment type and is beyond the scope of this manuscript.

To our knowledge there are currently no user-friendly solutions available for the allele-specific splitting of sequencing reads aligned to N-masked genomes. We sought to address this by creating SNPsplit, an easy-to-use tool for assigning allele-specifc reads. In its generic mode SNPsplit is not tied to any particular aligner and operates across several different experiment types including RNA-Seq, genomic DNA-alignments, DNA methylation (Bisulfite-Seq) and 3-D genome organisation (Hi-C). While a similar allele-specific functionality has been integrated into specialised applications, e.g. HiC-Pro ^10^, the unique capability to work with several different data types renders SNPsplit an ideal choice for correlation studies using allele-specific sequencing reads.

## Methods

### Implementation

SNPsplit is written in Perl and consists of three separate scripts that can be run individually on the command line. It takes alignment files in BAM/SAM format as input and further requires an annotation file containing the positions of all SNPs in the genome. SNPsplit determines for each aligned read whether it overlaps with a known SNP position and adds a tag to the alignment that indicates whether the read can be assigned to a specific allele or is unassignable. The reads are then sorted into different sub-files depending on the library type, i.e. single-end or paired-end, and the nature of the sample, e.g. RNA-Seq, BS-Seq or Hi-C.

### Generating N-masked genomes

As long as a SAM/BAM file that was aligned to an N-masked genome is provided as input SNPsplit should perform well regardless of how the N-masking itself was accomplished. Since there is an ever growing number of genomes and different SNP annotation files and file formats it would be too much to ask to provide a generally applicable way of constructing N-masked genomes that fits all cases.

**Figure 1.**
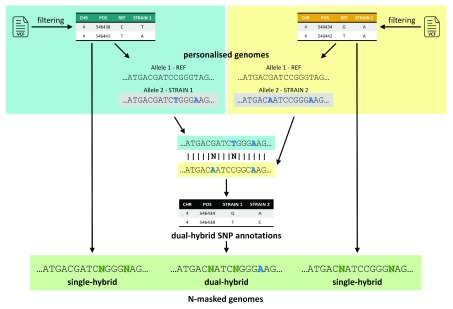
Generating N-masked or personalised genomes. Single-hybrid strains require SNP information/filtering from only one strain (Strain 1, blue box). SNP positions against the referene genome (REF) can either be masked by Ns (N-masked genome) or incorporated unmasked as full sequence (personalized genome). Dual hybrid strains require the SNP information/filtering also from a second strain (Strain 2, yellow box). The SNP information of Strain 1 and Strain 2 are then compared to create dual-hybrid SNP annotations (note that some positions where both strains had the same variation relative to the reference genome are no longer regarded as SNP and are now missing in the new annotations, e.g. A at position 546442). The dual-hybrid annotations are then used to N-mask SNP positions using the Strain 1 genome as new reference. The N-masked or personalised genomes for Strain 2 are technically not required to generate dual hybrid genomes but may be written out for convenience reasons.

We do however provide an automated solution to generate N-masked versions of the genome for all strains in the Mouse Genomes Project ( http://www.sanger.ac.uk/science/data/mouse-genomes-project). The genome preparation step supports the generation of single hybrid strains where one allele is the same as the mouse reference sequence (which is based on strain C57BL/6J, hereafter called Black 6) and one alternative allele, e.g. SPRET/EiJ. It also supports the generation of dual hybrid strains where both alleles are different from the Black 6 reference, e.g. CAST/EiJ and 129S1/SvImJ. At the time of writing the Mouse Genomes Project encompassed variation information for 36 different mouse strains; the SNP annotation data for all strains relative to Black 6 reference sequence may be found in the variant call format (VCF) file ‘mgp.v5.merged.snps_all.dbSNP142.vcf.gz’ (VCF v4.2; last modified 13 May 2015; download available at: ftp://ftp-mouse.sanger.ac.uk/current_snps/). The SNPsplit genome preparation first reads the SNP annotations for the strain in question from the VCF file and then constructs the N-masked genomes based on the Black 6 reference sequence using only high confidence homozygous positions. The process is slightly different for single or dual hybrid strains ( Figure 1).

***Single-Hybrid Strains*.** This generates a new genome sequence, with SNPs either N-masked or included as full sequence, where Allele 1 (or Genome 1) is the Black 6 reference and Allele 2 (or Genome 2) is the alternative strain.

1) The VCF file is read and filtered for high-confidence SNPs for the strain specified

2) The Black 6 reference genome is read into memory, and the filtered high-confidence SNP positions are incorporated either as N-masking (default) or full sequence (optional)

***Dual-Hybrid Strains*.** This generates a new genome sequence where neither allele is the Black 6 reference. SNPs can be either N-masked or included as full sequence, where Allele 1 (or Genome 1) is the strain specified as strain 1 and Allele 2 (or Genome 2) is the strain specified as strain 2.

1) The VCF file is read and filtered for high-confidence SNPs in strain 1

2) The Black 6 reference genome is read into memory, and the filtered high-confidence SNP positions are incorporated as full sequence and N-masking (optional)

3) The VCF file is read and filtered for high-confidence SNPs in strain 2

4) The filtered high-confidence SNP positions of strain 2 are incorporated as full sequence and N-masking (optional)

5) The SNP information of strain 1 and strain 2 relative to the Black 6 reference genome build are compared and a new Ref/SNP annotation is constructed whereby the new Ref/SNP information will be strain 1/strain 2

6) The full genome sequence of strain 1 is read into memory, and the high-confidence SNP positions between strain 1 and strain 2 are incorporated as full sequence and N-masking (optional)

The N-masked sequences (or sequences containing the full sequence SNPs) are written out in FASTA format and ready to be indexed with the alignment software of your choice. Alignments to N-masked genomes are not very different to regular mapping except that they require the aligner to support ambiguity DNA bases such as N. Software confirmed to be working for this approach include (but are not limited to) Bowtie2 ^11^, BWA ^12^, HISAT2 ^13^, STAR ^14^ or any tool wrapping one of these aligners.

Even though the automated genome preparation is optimised to work with the VCF file from the Mouse Genomes Project the process can be easily adapted to work with any other genome as long as the genotypes are known and well defined. The SNPsplit manual provides more detailed information about the SNP filtering from VCF files and which entries are required to make it work also with other genomes.

### Running SNPsplit on aligned files

SNPsplit operates in two stages which are run sequentially: I) read tagging and II) read sorting. Both steps generate detailed reports for record keeping.

**Stage I: Tagging** SNPsplit analyses reads for overlaps with known SNP positions for which it requires the mismatch position field (MD:Z:) in the SAM entry, and writes out a tagged BAM file in the same order as the original file. This process requires a list of all known SNP positions between the two different genomes (supplied as a SNP file) and works on a read-by-read basis.

Read tagging generally works as a multi-step process:

1. Determine the position(s) in the read that overlap genomic N(s)
2. Adjusting position for insertions/deletions
3. Determine equivalent genomic position
4. Determine if the SNP is present in the list of SNP positions, and if yes whether the position in the read was the Allele 1 or Allele 2 base

Depending on the collected SNP information the tagging module then determines whether a read can be assigned to a certain allele and appends an additional optional field ‘XX:Z:tag’ to the SAM entry of each read. The tag can be one of the following:

- UA - Unassigned
- G1 - Genome 1-specific (Allele 1, the reference)
- G2 - Genome 2-specific (Allele 2, the alternative strain)
- CF - Conflicting

Reads are considered unassignable (UA) if they do not overlap any known SNP position. Reads harbouring at least one SNP specific for both genomes at the same time are classified as conflicting (CF).

The determination of overlaps is geared to handle the CIGAR operations M (match to the reference), D (deletion in the read), I (insertion in the read) and N (skipped regions, used for splice mapping). Other CIGAR operations (see the SAM format specification for further details ^15^) are currently not supported. This means that SNPsplit requires reads to be a full match from end-to-end and thus soft-clipping (CIGAR operation: S), which may introduce artefactual alignments to poorly annotated regions in the genome ^16^ is not supported (see also section Use Cases for RNA-Seq below on how to avoid soft-clipping issues).

**Stage II: Sorting** The tagged BAM file is read in again and sorted into allele-specific files according to their XX:Z: tag. For paired-end or Hi-C experiments the combination of tags for both Read 1 and Read 2 are considered (see below for examples). Conflicting reads, or also disagreeing read-pairs for paired-end samples, are not printed out by default. The sorting process may also be run stand-alone on tagged BAM files to try out different sorting options (e.g. separating out paired-end and singleton alignments or enabling reporting of conflicting alignments).

### Operation

SNPsplit runs on any Linux-based operating system with Perl installed (tested using CentOS v6.2 and Perl v5.10.1). In addition, a functional version of SAMtools ^15^ (v0.1.18 or later) is required for handling of SAM/BAM files. Memory requirements depend directly on the genome size and the total number of heterozygous SNPs to be stored, but as a guideline 5–10 GB RAM should be sufficient to process data for most mouse strains.

## Use cases

### Standard genomic DNA alignments

SNPsplit is able to handle any kind of standard genomic alignment file irrespective of the method employed to generate the library as long as the CIGAR operation requirements are met (see Stage I: Tagging above). A non-exhaustive list of supported applications includes genome re-sequencing, histone or protein ChIP-Seq (chromatin immunoprecipitation sequencing) or ATAC-Seq (Assay for Transposase-Accessible Chromatin by sequencing).

**Figure 2.**
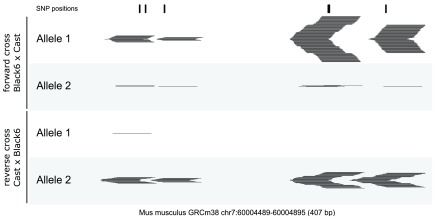
ChIP-Seq for the transcription factor ZFP57 identifies parental-origin allele-specific binding at the differentially methylated region (DMR) of the SNRPN locus. The binding of ZFB57 is methylation dependent and can be found exclusively on the maternal allele (genetic background of mother in forward cross: Black 6; mother in reverse cross: Cast). SNP positions were N-masked and used for allele-specific splitting of sequencing reads (shown as horizontal lines in black). Allele 1: Black 6 reference. Allele 2: Cast/EiJ strain (Cast). The area shown depicts the DMR only in part. Data taken from 17 (GEO accession: GSE55382).

A use case of ChIP-Seq for the transcription factor ZFP57 is shown in Figure 2 (data re-analysed from 17). Alignments to an N-masked reference genome were performed for reciprocal crosses between Black 6 and Cast/EiJ mice using Bowtie 2, followed by SNPsplit sorting. This process was able to identify allele-specific binding of ZFP57 to several different imprinting control regions in a parental origin-specific manner, exemplified for the SNRPN locus in Figure 2.

### RNA-Seq

In addition to standard linear alignments with or without small InDels, SNPsplit also handles spliced read alignments containing large gaps (CIGAR operation: N), such as reads spanning exon boundaries in RNA-Seq experiments. Spliced read aligners that have successfully been used for allele-specific alignments in conjunction with SNPsplit include Tophat ^18^, STAR ^14^ (Spliced Transcripts Alignment to a Reference) and HISAT2 ^13^. To work smoothly together with SNPsplit, HISAT2 and STAR require the user to disable soft-clipping which is performed by default (CIGAR operation: S), and STAR also needs to be instructed to print out the mismatch position (MD:Z:) field. More detailed instructions may be found in the SNPsplit User Guide.

### Hi-C

As a variant of the chromatin conformation capture assay Hi-C is a proximity-ligation based assay which allows the investigation of the three-dimensional structure of the genome by massively parallel sequencing ^19^. This is accomplished by measuring the frequency at which different parts of the genome sequence come into close physical contact. While standard Hi-C cannot discriminate whether an interacting fragment originated from the same or the other allele, allele-specific interaction maps can separate cis-allele from trans-allele interactions, thereby greatly improving the analysis of chromatin dynamics and gene regulation ^20, 21^.

The Hi-C mode of SNPsplit assumes that the input data is in the Hi-C format produced by the HiCUP pipeline ^22^, i.e. the input BAM files are by definition paired-end and Read 1 and Read 2 follow each other. It discriminates several additional read combinations to distinguish between cis- and trans-allele interactions:

- G1-G1
- G2-G2
- G1-UA
- G2-UA
- G1-G2
- UA-UA

For mixed allele groups such as G1-G2 there is no need to create the reverse group (G2-G1) since Hi-C interactions have no directionality. Again, read pairs containing at least one conflicting read (tag: CF) are not printed out by default, but this may be optionally enabled.

### Bisulfite-Seq

Bisulfite sequencing is a method to interrogate DNA methylation patterns using the chemical properties of sodium bisulfite to convert cytosines to uracil but leaving methylated cytosines largely unaffected.

The bisulfite mode of SNPsplit assumes that the input data has been processed with the bisulfite alignment tool Bismark ^23^. SNPsplit runs a quick check at the start of a run to see if the file provided appears to be a Bismark file, and sets the appropriate flags for bisulfite and/or paired mode automatically. Paired-end mode requires Read 1 and Read 2 of a pair to follow each other in consecutive lines so the BAM file will be sorted by read name if necessary.

**Utilisation of SNP positions and allele assignment of bisulfite treated reads** In contrast to the standard mode, C>T SNPs may not always be used for allele-specific sorting in a bisulfite setting since they could either be a genuine SNP or rather reflect the methylation state. Since the majority of known SNPs actually involves C to T transitions (due to spontaneous deamination of methylated CpG dinucleotides), the ability to assign aligned bisulfite treated reads is thus somewhat reduced compared to regular DNA-based alignments. The number of SNP positions that have been skipped because of this bisulfite ambiguity is documented in the report file.

Positions requiring special treatment include all of the following Allele 1/Allele 2 combinations: C/T or T/C for forward strand alignments and G/A or A/G for reverse strand alignments. These positions may however be used to assign opposing strand alignments since they do not involve C to T transitions directly. For that reason, the bisulfite call processing also extracts the bisulfite strand information from the alignments in addition to the basecall at the position involved. For any SNPs involving C positions that are not C to T SNPs both methylation states, i.e. C and T, are allowed to match the C position.

For SNPs which were masked by Ns in the genome no methylation call will have been performed during the alignment step, i.e. they will receive a ‘.’ (dot) in the methylation call string. This means that SNP positions themselves may be used for allele-sorting but do not participate in calling methylation. While this reduces slightly the number of total methylation calls it effectively eliminates the problem of assigning potentially incorrect methylation states to these positions.

**Figure 3.**
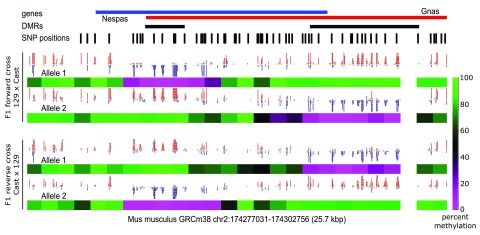
Allele-specific methylation at differentially methylated regions (DMRs) is maintained in a parent-of-origin specific way at the Gnas/Nespas locus. The upstream DMR is methylated exclusively on the paternal allele, while the more downstream DMR is methylated exclusively on the maternal allele in both forward (129 x Cast) and reverse crossed (Cast x 129) hybrid mice. SNP positions were N-masked and used for allele-specific sorting with SNPsplit. Allele 1: 129X1/SvJ reference (129). Allele 2: Cast/EiJ strain (Cast). Red or blue dots in the graph represent calls for methylated or unmethylated cytosines, respectively (CpG context only). The percentage methylation was determined for 2000 bp windows for the region shown using the Bisulfite Methylation Pipeline in Seqmonk ^25^ (default options). Data taken from 6, GEO accession: GSE33722.

To demonstrate the effectiveness of sorting bisulfite treated reads we reprocessed publicly available bisulfite sequencing data from reciprocal mouse crosses reported by Xie and colleagues ^6^ (GEO accession: GSE33722). First we generated a dual hybrid genome for 129X1/SvJ (129) (as near-enough relative we used the SNP annotations for strain 129S1/SvImJ) and Cast/EiJ (Cast) mice, and then aligned the data to the N-masked genome using Bismark (v0.16.1, default parameters, read trimming was performed using Trim Galore ^24^ v0.4.1, default parameters). The data was then processed with SNPsplit and all datasets for the F1 forward cross 129 (mother) x Cast (father), and F1 reverse cross Cast (mother) x 129 (father) were merged and analysed using SeqMonk (v.0.33.0 ^25^) ( Figure 3).

Whilst the majority of the genome shows very similar methylation levels on both alleles of the hybrid mice, this approach also allows the detection of allele-specific methylation events. This can be readily spotted at imprinted loci where one parental allele is fully methylated while the other remains completely unmethylated. The Gnas/Nespas locus in the mouse genome shows both a paternally methylated region (more upstream) and maternally methylated region (more downstream) where the allele-specific methylation pattern is maintained in a parent-of-origin dependent manner ( Figure 3). This demonstrates that the combination of bisulfite mapping and read sorting by SNPsplit is an effective tool to identify allele-specific methylation in diploid genomes.

## Summary

Analysing next-generation sequencing data in an allele-specific fashion holds the potential to uncover regulatory events or mechanisms that would otherwise be obscured in bulk data. SNPsplit is designed to enable researchers to quickly and easily perform allele-specific analysis of their sequencing data as long as the SNP genotypes of the organism in question are known. For hybrid mouse strains covered by the Mouse Genomes Project, SNPsplit offers an easy solution from generating N-masked genomes to allele-specific sorting of reads without requiring the user to possess excessive computational skills. SNPsplit is not tied to any specific application and indeed it has been used already to answer questions for a variety of different data types such as ChIP-Seq, RNA-Seq, Bisulfite-Seq and Hi-C. This gives SNPsplit the unique capability of bringing together allele-specific data including gene-expression, DNA methylation, genomic accessibility or architecture which holds great potential for studying genome regulation.

## Software availability

1. Software available from: http://www.bioinformatics.babraham.ac.uk/projects/SNPsplit/
2. Latest source code: https://github.com/FelixKrueger/SNPsplit
3. Archived source code as at time of publication: https://zenodo.org/record/55477#.V18PoDb93ww ^26^
4. Software license: GNU GPL v3 or later
