# Supplementary material for: SNPsplit: Allele-specific splitting of alignments between genomes with known SNP genotypes
Source: F1000Res. 2016 Jul 27;5:1479. Originally published 2016 Jun 23. [Version 2] doi: 10.12688/f1000research.9037.2 (PMC4934512; doi:10.12688/f1000research.9037.2)
Supplement: Peer review history

# Peer review: Referee response for version 2

**DOI:** 10.5256/f1000research.9980.r15257

**Published:** 2016-08-01

**Authors:** Andrew Keniry

**Affiliations:** 1 Walter and Eliza Hall Institute of Medical Research, Parkville, Vic, Australia

I have read this submission. I believe that I have an appropriate level of expertise to confirm that it is of an acceptable scientific standard.

# Peer review: Referee response for version 2

**DOI:** 10.5256/f1000research.9980.r15258

**Published:** 2016-07-27

**Authors:** Prasoon Agarwal

**Affiliations:** 1 Division of Clinical Immunology, Department of Laboratory Medicine, Karolinska Institutet, Huddinge, Sweden

I have read this submission. I believe that I have an appropriate level of expertise to confirm that it is of an acceptable scientific standard.

# Peer review: Referee response for version 1

**DOI:** 10.5256/f1000research.9725.r14556

**Published:** 2016-07-08

**Authors:** Prasoon Agarwal

**Affiliations:** 1 Division of Clinical Immunology, Department of Laboratory Medicine, Karolinska Institutet, Huddinge, Sweden

This article by Krueger F *et al*. describes in detail a new software SNPsplit which is capable of sorting reads or read–pairs in the allele specific modus that covers know single nucleotide polymorphic (SNP), or single nucleotide variation (SNV) locations. For sorting the reads the inputs to the software are a ‘N’ masked genome, created using known SNP or SNV locations, to which the reads are aligned using any available aligner and a VCF file containing the positions of all SNPs. The authors claim that this is the only user-friendly solution available for the allele-specific splitting of sequencing reads aligned to N-masked genomes. Overall the manuscript is very well written. However, I have some minor queries regarding the software performance:

1. Masking known SNP positions in the genome sequence eliminated the reference bias but, in case of heterozygous SNPs there could be a chance of having significant bias toward higher mapping rates of the allele in the reference sequence, is there any provision in the software to remove this noise and bias from mapped reads? This kind of bias can lead to false signal of allelic imbalance. 
2. It is stated that the software can construct the N-masked genomes, so is it restricted to mouse alone or can be extended in case of humans or other species?
3. Can SNPsplit be used to split reads for indels and deletions?

I have personally used the software for ATAC-seq data from patients and it works perfectly fine. I have not used the genome builder module of the software. The data looks perfect in the UCSC genome browser for the ‘Allele 1’ or ‘Allele 2’ and the unassigned. Overall I felt it is a very user friendly software available.

I have read this submission. I believe that I have an appropriate level of expertise to confirm that it is of an acceptable scientific standard.

# Response

**Published:** 2016-07-18

**Authors:** Felix Krueger

**Affiliations:** The Babraham Institute, UK

We would like to thank the reviewer for their kind and approving comments about SNPsplit. Some specific comments may be found below.

**Masking known SNP positions in the genome sequence eliminated the reference bias but, in case of heterozygous SNPs there could be a chance of having significant bias toward higher mapping rates of the allele in the reference sequence, is there any provision in the software to remove this noise and bias from mapped reads? This kind of bias can lead to false signal of allelic imbalance.**

 SNPsplit is really designed for mapping against genomes with clean parental genotypes; this is also why the genome preparation step is very strict and only uses high quality homozygous parental SNPs (see the new section on this in the SNPsplit manual). If the SNPs are heterozygous already in one or even both of the parental strains we propose that these positions could be masked by Ns for the mapping step, but not be used at all for the allele-specific read assignment as such. For the allele-specific splitting, i.e. the file supplied to SNPsplit with --snp_file, only homozygous high quality SNP positions should be used.

**It is stated that the software can construct the N-masked genomes, so is it restricted to mouse alone or can be extended in case of humans or other species?**

 The SNPsplit approach does in theory work on any N-masked genome irrespective of the species. The preparation is currently optimised to work the VCF file provided by the Mouse Genomes Project but as long as the VCF file conforms to the same standards it should also work for other genomes. If the VCF file looks different, e.g. you got the file from a collaborator and it came without header lines or format description one would have to adapt the relevant section(s) in the preparation script. We have now added a section to the SNPsplit User Guide that explains in more detail the basis of SNP filtering and which parameters are needed to run properly. We hope that this will help generating N-masked genomes when the SNP data looks different than the file provided by Mouse Genomes Project.

**Can SNPsplit be used to split reads for indels and deletions?**

 SNPsplit supports the processing of reads that contain indels, but indels themselves are not used to sort reads to different genomes at the current time. The reason for this is mainly that indel annotations are often more tricky to come by and alignments over indels (e.g. at the ends of reads) are notoriously more difficult. There are currently no plans to extend the functionality to include indels, but we might look into this in the future if the demand arises.

# Peer review: Referee response for version 1

**DOI:** 10.5256/f1000research.9725.r14557

**Published:** 2016-07-01

**Authors:** Nicolas Servant

**Affiliations:** 1 Institut Curie, Paris, France

This manuscript by Krueger and collaborators describes SNPsplit, an alignment sorter for allele specific analysis. SNPsplit is designed to work on N-masked alignment and provides additional utilities to generate the appropriate reference.

 The main interest of SNPsplit is its ability to operate across different experiment types and alignment software. It therefore allows to analyse and to integrate in an allele specific and unbiased manner heterogeneous dataset.

 The manuscript is well written, and is divided in two main parts. The first part presents the software and its implementation and the second part, presents user cases.

 Other than my few comments below, I am happy with the manuscript and want to note that I have successfully downloaded and used SNPsplit in the context of several projects.

1. The way the N-masked genome is generated based on SNPs information is not that easy to understand for non expert users. I would recommend a figure to help in understanding this point.
  In the context of Dual-Hybrid strain, the interest of first generating the strain 1 (S1) and the strain 2 (S2) genomes is not clear to me.
  If I'm correct, the final masked genome will be generated from the S1 fasta reference only (unmasked). If we use a simple example with a SNP which is Bl6=A, S1=T, S2=T, using this strategy will avoid a mismatch at this position, compared to the strategy of masking only heterozygous SNPs between S1/S2 on the reference (Bl6) genome. But what is the interest of generating the S2 reference genome (step 4) ? Using S1 or S2 should be enough?
2. How paired-end sequencing is managed in practice ? The authors present an application with Hi-C data but is it the same for any NGS application?
3. The authors present SNPsplit in the context of the Mouse Genome Project. I'm wondering how difficult it would be to transpose it to any genome and organism as long as the genotype is known. A few words about that in the manuscript would be interesting.
4. In the Introduction, the authors mentioned the WASP software[7]. To avoid any misunderstanding with the parental genome strategy, I would suggest something like; “A more recent improvement aims to reduce mapping biases by first aligning reads on the reference genome, then realigning reads that overlap SNP positions in all possible allele combinations and keeping only reads that align to the same position regardless their genotypes - this reduces bias, but is computationally complex”
5. At the end of the introduction, the authors mentioned that “SNPsplit is not tied to any particular aligner” which is not exactly true as the authors explain later that “SNPsplit can be used as long as the CIGAR operation requirements are met”
6. Typo:
  “The determination of overlaps is geared” in **Stage I: Tagging**
  Reference 26 and 27 doesn't seem to be used in the text

I have read this submission. I believe that I have an appropriate level of expertise to confirm that it is of an acceptable scientific standard.

# Response

**Published:** 2016-07-18

**Authors:** Felix Krueger

**Affiliations:** The Babraham Institute, UK

Many thanks for the constructive review and the thoughtful suggestions on how to improve the manuscript. Please find our point-by-point responses below.

**The way the N-masked genome is generated based on SNPs information is not that easy to understand for non expert users. I would recommend a figure to help in understanding this point.**

 We have now added a new Figure 1 that aims to explain the process of generating single or dual hybrid N-masked genomes in more detail. We feel that this new figure makes the whole process substantially easier to understand, many thanks for suggestion!

**In the context of Dual-Hybrid strain, the interest of first generating the strain 1 (S1) and the strain 2 (S2) genomes is not clear to me.**

**If I'm correct, the final masked genome will be generated from the S1 fasta reference only (unmasked). If we use a simple example with a SNP which is Bl6=A, S1=T, S2=T, using this strategy will avoid a mismatch at this position, compared to the strategy of masking only heterozygous SNPs between S1/S2 on the reference (Bl6) genome. But what is the interest of generating the S2 reference genome (step 4) ? Using S1 or S2 should be enough?**

 Technically it would be sufficient to use the Strain 1 unmasked genome as new reference and then use the SNP annotations of both strains to compute a new Strain1/Strain2 SNP annotation file (see also the new Figure 1). Since writing out a new genome only takes a few seconds and it might be of potential use for other projects we do also write out versions of Strain 2, it can always be removed later if desired.

**How paired-end sequencing is managed in practice? The authors present an application with Hi-C data but is it the same for any NGS application?**

 We do mention in the manuscript that the paired-end mode uses both ends for the genome-specific assignments even though it is not as complicated as the Hi-C mode. The SNPsplit User Guide already contains more detailed information about paired-end file handling and also provided paired-end sorting reports to illustrate this, so we would kindly refer the user to the manual rather than adding an extra section here.

**The authors present SNPsplit in the context of the Mouse Genome Project. I'm wondering how difficult it would be to transpose it to any genome and organism as long as the genotype is known. A few words about that in the manuscript would be interesting.**

 Indeed, as long as the genotypes are well defined and the list of SNP positions is known the genome preparation should also work well with any other genome (see also the reply to Prasoon Agarwal’s comment). To enable this process we have added a new section to the SNPsplit User Guide that explains the SNP filtering and processing in more detail, and we have added a short paragraph about this to the manuscript.

**In the Introduction, the authors mentioned the WASP software[7]. To avoid any misunderstanding with the parental genome strategy, I would suggest something like; “A more recent improvement** **aims to reduce mapping biases by first aligning reads on the reference genome, then realigning reads that overlap SNP positions in all possible allele combinations and keeping only reads that align to the same position regardless their genotypes - this reduces bias, but is computationally complex”**

 We have changed this sentence to make it clearer.

**At the end of the introduction, the authors mentioned that “SNPsplit is not tied to any particular aligner” which is not exactly true as the authors explain later that “SNPsplit can be used as long as the CIGAR operation requirements are met”**

 At least in its generic mode SNPsplit is not tied to any specific aligner, but this does not necessarily mean that every single option of any aligner will be supported. To this end, SNPsplit was initially designed to work with Bowtie2 and Tophat, but we have later also seen other software such as STAR or HISAT2 work fine as well. Aligner-specific comments can be found in the SNPsplit User guide.

**Reference 26 and 27 doesn't seem to be used in the text**

 Thanks for spotting this, we have now removed these references from the bibliography.

# Peer review: Referee response for version 1

**DOI:** 10.5256/f1000research.9725.r14558

**Published:** 2016-07-01

**Authors:** Andrew Keniry

**Affiliations:** 1 Walter and Eliza Hall Institute of Medical Research, Parkville, Vic, Australia

Krueger and Andrews report SNPsplit, a tool for sorting mapped next generation sequencing reads into separate files depending on the allelic origin of the read. The process requires genetic heterogeneity between the alleles such that the reads can be assigned to a particular allele based on known SNP positions. Such a tool allows for allele specific analysis and becomes useful for studies on phenomena such as genomic imprinting, allelic imbalance and X-chromosome inactivation. SNPsplit improves on current methods for analysis of allele specific sequencing reads by providing built in tools for the analysis of bisulfite and HiC data, which are otherwise more complicated to analyse. A tool for creating an N-masked genome is also provided, which overcomes mapping bias towards the reference genome.

 To date I have used SNPsplit to process data from RNA-seq, bisulfite-seq, transcription factor ChIP-seq and histone ChIP-seq. I have not tested the built in N-masked genome creator. SNPsplit has proven to be easy to use and very stable in my run environment. Typically, a sample is processed in approximately 3 hours depending on read depth. As far as I can tell, by assessing known imprinted genes and the silent female X chromosome, SNPsplit does an excellent job of assigning reads to the correct genome, with known phenomena appearing as expected. The built in option for analysis of bisulfite data works very well on reads mapped with the bismark program.

 I’ve found SNPsplit to work very well for all the data types I have used it for: RNA-seq, bisulfite-seq, transcription factor ChIP-seq and histone ChIP-seq. It should be noted however that due to the requirement for a SNP to be present in the read, assignable reads from narrow transcription factor ChIP-seq peaks can be sparse and some peaks may not assignable at all. This is an unavoidable limitation, and the authors show successful assignment of transcription factor ChIP-seq reads in Figure 2, however this is perhaps something researchers should consider. Reads deriving from broad histone ChIP-seq peaks suffer no such limitation.

 I have no issues with the performance of SNPsplit, and neither the accuracy of how the tool is presented in the paper. Perhaps the authors could include some details that will aid researchers in experimental design?  For example, for a typical SNP density what percentage of reads would be assigned to each genome, unassigned and conflicting? This would help researchers in estimating required read depths for their particular question. Could the authors also explain how vcf files are filtered for high confidence SNPs when preparing the N-masked genome?  A discussion of post processing techniques for the removal of incorrectly annotated SNPs would also be beneficial. Perhaps the benefit of longer read length for assigning reads to a particular genome could also be mentioned?

 In summary, SNPsplit will prove to be a very useful tool for the analysis of epigenomic data from next generation sequencing experiments.

I have read this submission. I believe that I have an appropriate level of expertise to confirm that it is of an acceptable scientific standard.

# Response

**Published:** 2016-07-18

**Authors:** Felix Krueger

**Affiliations:** The Babraham Institute, UK

We would like to thank the reviewer for their kind and positive feedback. Please find our specific replies below.

**Could the authors also explain how vcf files are filtered for high confidence SNPs when preparing the N-masked genome?**

 We have expanded the SNPsplit User Guide considerably to now include a detailed section on how the SNP filtering is accomplished and which of the parameters are required for the process to work with other VCF files. We hope that this will make the process easier to understand and allow adapting the procedure for other genomes as well.

**Perhaps the authors could include some details that will aid researchers in experimental design?  For example, for a typical SNP density what percentage of reads would be assigned to each genome, unassigned and conflicting? This would help researchers in estimating required read depths for their particular question. A discussion of post processing techniques for the removal of incorrectly annotated SNPs would also be beneficial. Perhaps the benefit of longer read length for assigning reads to a particular genome could also be mentioned?**

 It would be undoubtedly helpful to provide some more guidance about experimental design, however we are not sure that this should be added in the context of software manuscript. As a general guideline the percentage of reads that can be assigned allele specifically increases proportionally with the number of heterozygous SNPs present between two strains, and increasing the read length also increases the chances to hit a SNP. Furthermore paired-end data can be assigned with a much rate than single-end reads. The SNPsplit User Guide contains a few example reports to help users to get an idea about typical values. We also feel that post-processing techniques probably warrant more discussion than just being briefly mentioned here, a good paper to read to get started is also cited in the manuscript (Castel et al.).
